# NEAT1–SOD2 Axis Confers Sorafenib and Lenvatinib Resistance by Activating AKT in Liver Cancer Cell Lines

**DOI:** 10.3390/cimb45020071

**Published:** 2023-01-29

**Authors:** Hiroyuki Tsuchiya, Ririko Shinonaga, Hiromi Sakaguchi, Yutaka Kitagawa, Kenji Yoshida

**Affiliations:** 1 Division of Molecular and Genetic Medicine, Graduate School of Medicine, Tottori University, 86 Nishi-cho, Yonago 683-8503, Japan; 2 Department of Radiation Oncology, Tottori University Hospital, 86 Nishi-cho, Yonago 683-8503, Japan

**Keywords:** NEAT1, liver cancer cell line, drug resistance, SOD2, MAPK, AKT

## Abstract

This study investigated the effects of a long noncoding RNA, nuclear paraspeckle assembly transcript 1 (NEAT1) variant 1 (NEAT1v1) on drug resistance in liver cancer cell lines. NEAT1 knockdown activated mitogen-activated protein kinase (MAPK) signaling pathways, including MAPK kinase (MEK)/extracellular signal-regulated kinase (ERK), but suppressed AKT. Moreover, NEAT1 knockdown sensitized liver cancer cells to sorafenib and lenvatinib, both clinically used for treating hepatocellular carcinoma, whereas it conferred resistance to an AKT-targeted drug, capivasertib. NEAT1v1 overexpression suppressed MEK/ERK and activated AKT, resulting in resistance to sorafenib and lenvatinib and sensitization to capivasertib. Superoxide dismutase 2 (SOD2) knockdown reverted the effects of NEAT1v1 overexpression on the sensitivity to the molecular-targeted drugs. Although NEAT1 or SOD2 knockdown enhanced endoplasmic reticulum (ER) stress, concomitant with the suppression of AKT, taurodeoxycholate, an ER stress suppressor, did not restore AKT activity. Although further in vivo and clinical studies are needed, these results suggested that NEAT1v1 switches the growth modality of liver cancer cell lines from MEK/ERK-dependent to AKT-dependent mode via SOD2 and regulates sensitivity to the molecular-targeted drugs independent of ER stress.

## 1. Introduction

Liver cancer is one of the malignant tumors with high mortality, making it the third leading cause of cancer-related deaths worldwide [1]. Hepatocellular carcinoma (HCC) is the most prevalent subtype of liver cancer. HCC in the early to middle stages can be treated with surgical resection or locoregional therapies, including radiofrequency ablation and transarterial chemoembolization [2]. In contrast, systemic chemotherapy is indicated for patients with advanced HCC who are not eligible for surgery or locoregional therapies [2].

Sorafenib and lenvatinib are multityrosine kinase inhibitors clinically used to treat HCC, although their preferential targets differ. The primary target kinases of sorafenib are vascular endothelial growth factor receptor (VEGFR), platelet-derived growth factor receptor (PDGFR), FMS-like tyrosine kinase 3 (FLT3), c-KIT, RAF1, and B-RAF, thereby suppressing mainly the mitogen-activated protein kinase (MAPK) cascade, including MAPK kinase (MEK) and extracellular signal-regulated kinase (ERK) [3,4]. Lenvatinib preferentially inhibits the tyrosine kinase activities of VEGFR, PDGFR, RET, c-KIT, and fibroblast growth factor receptor (FGFR), leading to the suppression of their downstream signaling pathways, including the MEK/ERK and phosphatidylinositol 3-kinase (PI3K)/AKT pathways [4,5,6]. By virtue of their inhibitory properties, both suppress tumor cell proliferation and neoangiogenesis and eventually prolong the survival of patients with advanced HCC [2]. However, the efficacy is limited in part by drug resistance [7].

It was demonstrated that alternative activation of P38MAPK supported RAF-independent activation of the MEK/ERK pathway in the presence of sorafenib and was required to acquire sorafenib resistance in the mouse HCC model [8]. AKT activation also induced sorafenib resistance in HCC by suppressing sorafenib-induced autophagic cell death [9,10] or by inducing forkhead box M1 expression via a transcription factor, activator protein 1 [11]. AKT-targeted drugs, MK2206 and ipatasertib, reversed sorafenib resistance in HCC cells [9,12]. Hepatocyte growth factor activated AKT through c-MET and concomitantly attenuated the antitumor effects of lenvatinib in HCC cells highly expressing c-MET [13]. Likewise, the suppression of phosphatase and tensin homologs by proprotein convertase subtilisin/kexin type 9 activated AKT, leading to the acquisition of sorafenib resistance in HCC [14].

A long noncoding RNA, nuclear paraspeckle assembly transcript 1 (NEAT1), is required for the formation of paraspeckle [15]. The NEAT1 gene is expressed as two variant isoforms: NEAT1v1 (3.8 kb in length in humans) and NEAT1v2 (22.7 kb). Both of these are transcribed from the same nucleotide position but have different sites of transcriptional termination [15]. NEAT1v2 is required for the formation of paraspeckle, which is a nuclear substructure found in most cultured cells. NEAT1v1 is also incorporated into paraspeckles, but it also exists in “microspeckle”, outside of paraspeckles [16], suggesting that NEAT1v1 has intrinsic functions independent of NEAT1v2. We have previously demonstrated that NEAT1v1, but not NEAT1v2, is involved in the maintenance of liver cancer stem cells and confers radioresistance to liver cancer cell lines [17,18]. It confers radioresistance to liver cancer cell lines by inducing mitophagy via superoxide dismutase 2 (SOD2) and γ-aminobutyric acid A receptor-associated protein (GABARAP) [19]. Moreover, NEAT1 induced sorafenib resistance in HCC cells by activating the c-MET/AKT pathway via microRNA (miR)-335 [20] or promoting autophagy via the miR-204/autophagy-related 3 axis [21]. However, the relationship between NEAT1 and lenvatinib has not been investigated.

This study investigated the effects of NEAT1v1 on the sensitivity of liver cancer cell lines to sorafenib and lenvatinib and found that NEAT1v1 confers resistance to these drugs by activating AKT via superoxide dismutase 2 (SOD2) on the one hand, and NEAT1v1 concomitantly sensitizes cells to an AKT-targeted drug, capivasertib, on the other hand. In agreement with these findings, NEAT1v1 activates AKT while suppressing MAPK signaling molecules, including MEK/ERK, P38MAPK, and c-Jun N-terminal kinase (JNK). Although NEAT1 or SOD2 knockdown increased ER stress, concomitant with AKT suppression, taurodeoxycholate (TUDC), an ER stress suppressor, did not restore AKT activity. These results suggested that the NEAT1v1–SOD2 axis promotes AKT-dependent growth independent of ER stress. Moreover, AKT-targeted drugs are promising as another therapeutic option for treating advanced HCC.

## 2. Materials and Methods

All resources used in the present study are summarized in Table S1.

### 2.1. Cell Culture

Human liver cancer cell lines HLE and HuH6 were purchased from the Japanese Collection of Research Bioresources Cell Bank (Osaka, Japan) and maintained in Dulbecco’s Modified Eagle’s Medium (Nissui Pharmaceutical, Tokyo, Japan) supplemented with 10% inactivated fetal bovine serum (Sigma-Aldrich, St. Louis, MO, USA). HLF and HuH6 cells overexpressing human NEAT1v1 were reported previously [16,17]. In brief, HLF and HuH6 cells were stably transfected with pcDNA6-hNEAT1v1-AcGFP [18]. Following blasticidin (Kaken Pharmaceutical, Tokyo, Japan) selection, AcGFP-positive cells were sorted by flow cytometry.

### 2.2. Adenovirus Construction

The construction of adenovirus vectors was previously reported [17,18,19]. In brief, shNT, shNEAT1a/b, or shSOD2a/b were ligated into BsaI-digested pENTR/U6-AmCyan1 with Ligation High version 2 (Toyobo, Osaka, Japan). These oligo DNAs are shown in Table S1. The shRNA and AmCyan1-expressing cassettes were transferred by the LR reaction to pAd/BLOCK-iT-DEST (Thermo Fisher Scientific, Waltham, MA, USA). Adenovirus vectors were constructed by transfecting adenovirus plasmid DNA with Lipofec-tAMINE2000 into 293A cells (Thermo Fisher Scientific) according to the manufacturer’s protocol. Adenovirus titer was determined by the infectious genome titration protocol [22]. Adenovirus transduction was performed at 200 multiplicities of infection 24 h after seeding.

### 2.3. Drug Treatment and WST Assay

Cells were treated with sorafenib (Adipogen Life Sciences, San Diego, CA, USA), lenvatinib (Toronto Research Chemicals, Toronto, ON, Canada), and capivasertib (Adooq Bioscience, Irvine, CA, USA) at the concentrations indicated in the figures, or DMSO as the control for 48 h in a 96-well plate. In the knockdown experiments, adenovirus vectors were transduced 48 h before drug treatment. After treatment, the WST assay was performed with Cell Counting Kit-8 (Dojindo, Kumamoto, Japan) according to the manufacturer’s protocol.

### 2.4. TUDC Treatment

Cells were seeded in a 3.5 cm dish for 24 h. TUDC (Nacalai Tesque, Kyoto, Japan) was added to cells at a concentration of 200 mM. The adenovirus vectors were transduced at the same time as the TUDC treatment. After 48 h incubation, mRNA or protein was recovered from cells.

### 2.5. Reverse Transcription-Quantitative PCR (RT-qPCR) and Western Blot Analysis

RT-qPCR and Western blot analysis were performed as reported previously [17,18,19]. mRNA and protein samples were prepared 48 h after seeding, drug treatment, or adenovirus transduction. The primers used for RT-qPCR are summarized in Table S2. An amount of 0.2–1 µg of total RNA was used for the RT reaction, while an amount of 20–100 µg of protein was used for Western blot analysis. β-Actin was used as an internal control for calculating the relative mRNA expression levels. The antibodies for Western blot analysis were as follows: AKT (#9272), P-AKT (S473; #9271), P-AMP-activated protein kinase α (AMPKα) (T172; D79.5E; #4188), P-eukaryotic translation initiation factor 2α (EIF2α) (S51; #9721), P-ERK1/2 (Y202/204; #9101), inositol-requiring enzyme 1α (IRE1α) (14C10; #3294), JNK (#9252), P-JNK (T183/Y185; #9251), P-MEK1/2 (Ser217/221; 41G9;#9154), P-mammalian target of rapamycin (mTOR) (S2448; #2971), P38 (#9212), and P-P38 (T180/Y182; #9211) from Cell Signaling Technology (Danvers, MA, USA); AMPKα1/2 (D-6; sc-74461), activating transcription factor 4 (ATF4) (B-3; sc-390063), ATF6α (F-7; sc-166659), EIF2α (D-3; sc-133132), ERK1/2 (C-9; sc-514302), glyceraldehyde 3-phosphate dehydrogenase (GAPDH) (G-9; sc-365062), MEK1/2 (9G3; sc-81504), PRKR-like ER kinase (PERK) (B-5; sc-377400), β-tubulin (βTUB) (G-8; sc-55529), and X-box binding protein 1 (XBP1) (F-4; sc-8015) from Santa Cruz Biotechnology (Santa Cruz, CA, USA); and P-IRE1(S724; EPR5253; ab124945) from Abcam (Cambridge, MA, USA). After transferring proteins to polyvinylidene difluoride membranes, the membranes were horizontally cut and probed with the antibodies. GAPDH and βTUB (for total and unphosphorylated proteins) and total proteins (for corresponding phosphorylated proteins) were used for internal control.

### 2.6. Statistical Analysis

Three or more independent samples for each experiment were analyzed, and all experimental values were expressed as the mean ± standard deviation. The differences between the two groups were assessed by Student’s *t*-test. Multiple comparisons were made by Dunnett’s or Tukey’s tests as indicated. *p* < 0.05 was considered statistically significant.

## 3. Results

### 3.1. NEAT1 Knockdown Sensitizes Liver Cancer Cells to Sorafenib and Lenvatinib

**Figure 1:**
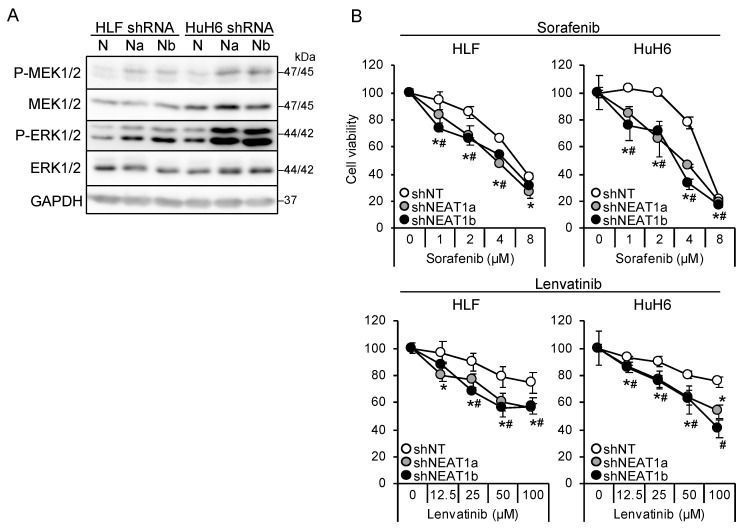
NEAT1 knockdown induces sorafenib and lenvatinib resistance. (**A**) Representative Western blot images for the indicated proteins. GAPDH is shown as an internal control. HLF and HuH6 cell lines were transduced with adenoviruses expressing nontargeting shRNA [shNT (N)] or NEAT1-specific shRNAs [shNEAT1a (Na) and shNEAT1b (Nb)] for 48 h. (**B**) Viabilities of HLF and HuH6 cells treated with sorafenib or lenvatinib at the concentrations indicated in the figure for 48 h relative to cells treated with dimethyl sulfoxide (DMSO; 100%). Cells were transduced with adenoviruses expressing shNT, shNEAT1a, and shNEAT1b 48 h before drug treatment. * *p* < 0.05 vs. shNT vs. shNEAT1a; # *p* < 0.05 shNT vs. shNEAT1b (Dunnett’s test; *n* = 4).

Two NEAT1-specific short hairpin RNAs (shRNAs; shNEAT1a and shNEAT1b) previously constructed [17,18] were used in this study. Both shRNAs activated MEK and ERK in liver cancer cell lines (HLF and HuH6; Figure 1A). Moreover, NEAT1 knockdown also activated P38MAPK and JNK (Figure S1A). After treatment with sorafenib and lenvatinib, the viability of cells knocked down for NEAT1 decreased significantly more than that of cells transduced with nontargeting shRNA (shNT; Figure 1B), suggesting that NEAT1 knockdown sensitized cells to these drugs. Although the activation of MEK and ERK by NEAT1 knockdown was higher in HuH6 cells than in HLF cells, the sensitization was similar between the cell lines.

### 3.2. NEAT1 Knockdown Confers Resistance against an AKT-Targeted Drug, Capivasertib

**Figure 2:**
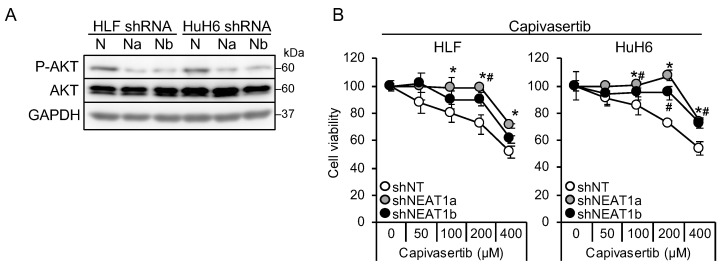
NEAT1 knockdown induces resistance against an AKT-targeted drug, capivasertib. (**A**) Representative Western blot images for the indicated proteins. GAPDH is shown as an internal control. HLF and HuH6 cell lines were transduced with adenoviruses expressing non-targeting shRNA [shNT (N)] or NEAT1-specific shRNAs [shNEAT1a (Na) and shNEAT1b (Nb)] for 48 h. (**B**) Viabilities of HLF and HuH6 cells treated with capivasertib at the concentrations indicated in the figure for 48 h relative to cells treated with DMSO (100%). Cells were transduced with adenoviruses expressing shNT, shNEAT1a, and shNEAT1b 48 h before drug treatment. * *p* < 0.05 vs. shNT vs. shNEAT1a; # *p* < 0.05 shNT vs. shNEAT1b (Dunnett’s test; *n* = 4).

It is suggested that AKT activation is one of the mechanisms underlying sorafenib resistance in HCC [9,10]. Thus, this study investigated whether AKT activity was affected by NEAT1 knockdown. AKT phosphorylation decreased in liver cancer cell lines knocked down for NEAT1 (Figure 2A). In contrast to MEK and ERK, the activation of AKT by NEAT1 knockdown was similar between HLF and HuH6 cell lines. Representative targets of AKT, mTOR, and AMPK were also examined, but their phosphorylation statuses were not changed (Figure S1A). In contrast to sorafenib and lenvatinib, cells showed resistance to an ATP-competitive AKT inhibitor, capivasertib (Figure 2B). These results suggested that NEAT1 knockdown endows liver cancer cells with resistance to AKT-targeted drugs.

### 3.3. NEAT1v1 Plays a Role as a Molecular Switch of Cell Growth Modality

**Figure 3:**
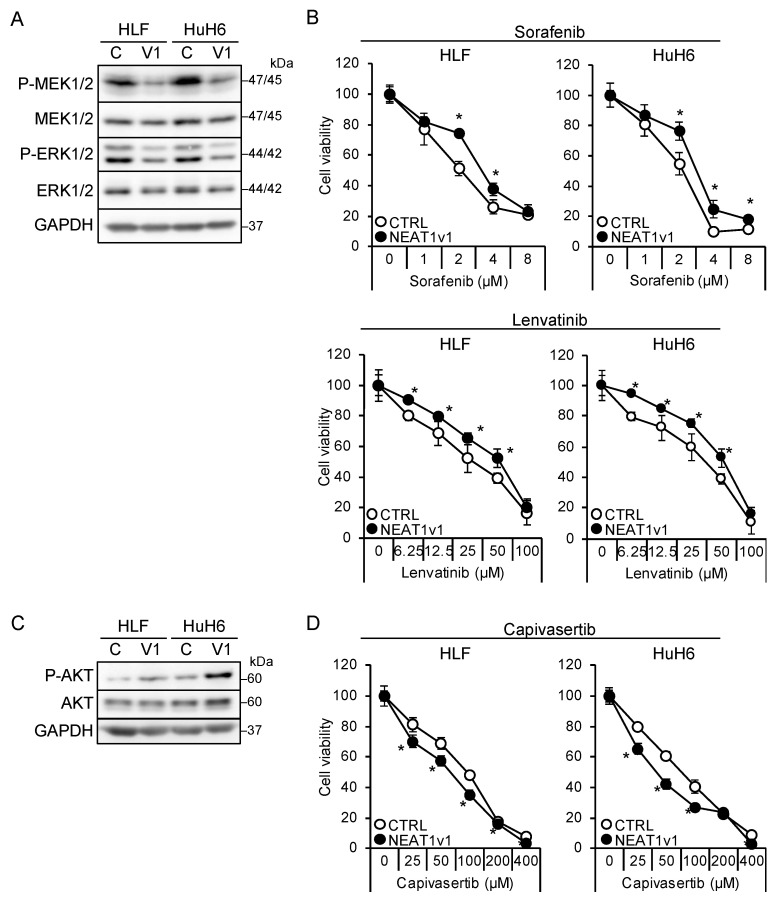
NEAT1v1 plays a role as a molecular switch of cell growth modality. (**A**,**C**) Representative Western blot images for the indicated proteins in control (**C**) or NEAT1v1-overexpressing (V1) cells. GAPDH is shown as an internal control. (**B**,**D**) Viabilities of control (CTRL) or NEAT1v1-overexpressing (NEAT1v1) cells treated with sorafenib (**B**), lenvatinib (**B**), or capivasertib (**D**) at the concentrations indicated in the figure for 48 h relative to cells treated with DMSO (100%). * *p* < 0.05 vs. CTRL (Student’s *t*-test; *n* = 4).

The shorter isoform, NEAT1v1, but not the longer one, NEAT1v2, is sufficient to induce cancer stemness and radioresistance [17,18,19]. Thus, liver cancer cell lines overexpressing NEAT1v1 [18,19] were used to examine whether NEAT1v1 could determine drug sensitivity. NEAT1v1 overexpression suppressed MEK and ERK, whereas P38MAPK and JNK phosphorylation was not affected (Figure 3A and Figure S1B). In contrast to knockdown, the activation of MEK and ERK by NEAT1 overexpression was similar between HLF and HuH6 cell lines. In addition, sensitivity to sorafenib and lenvatinib significantly decreased (Figure 3B). In contrast, NEAT1v1 activated AKT and sensitized cells to capivasertib (Figure 3C,D), whereas mTOR and AMPK were not activated (Figure S1B). AKT activation and sensitization to capivasertibe were more prominent in HuH6 cells than in HLF cells. These results suggested that NEAT1v1 is a molecular switch of growth modalities from MEK/ERK- to AKT-dependent growth in liver cancer cells.

### 3.4. NEAT1v1 Regulates the Growth Modality of Liver Cancer Cells through SOD2

**Figure 4:**
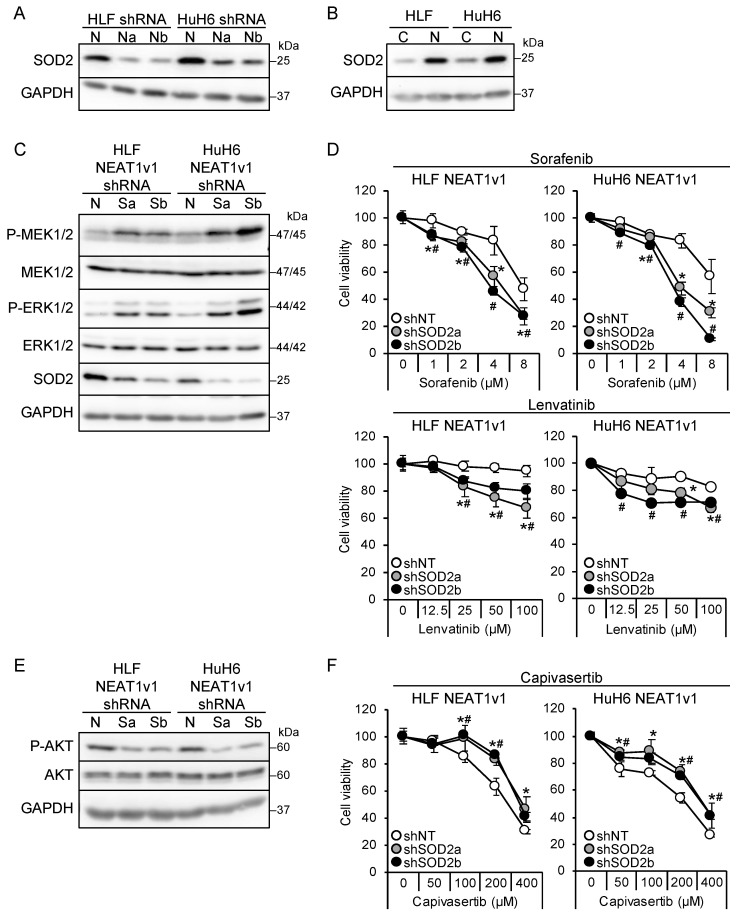
NEAT1v1 regulates cell growth modality through SOD2. (**A**) Representative Western blot images for SOD2 expression in HLF and HuH6 cells transduced with adenoviruses expressing shNT (N), shNEAT1a (Na), or shNEAT1b (Nb) for 48 h. GAPDH is shown as an internal control. (**B**) Representative Western blot images for SOD2 expression in control (**C**) or NEAT1v1-overexpressing (V1) HLF and HuH6 cells. (**C**,**E**) Representative Western blot images for the indicated proteins in NEAT1v1-overexpressing cells transduced with adenoviruses expressing shNT (N) or SOD2-specific shRNAs [shSOD2a (Sa) and shSOD2b (Sb)] for 48 h. (**D**,**F**) Viabilities of cells treated with sorafenib (**D**), lenvatinib (**D**), or capivasertib (**F**) at the concentrations indicated in the figure for 48 h relative to cells treated with DMSO (100%). NEAT1v1-overexpressing cells were transduced with adenoviruses expressing shNT, shSOD2a, and shSOD2b 48 h before drug treatment. * *p* < 0.05 vs. shNT vs. shSOD2a; # *p* < 0.05 shNT vs. shSOD2b (Dunnett’s test; n = 4).

We found that NEAT1 knockdown and NEAT1v1 overexpression resulted in the downregulation and upregulation, respectively, of SOD2 in liver cancer cells (Figure 4A,B). SOD2 knockdown in liver cancer cell lines overexpressing NEAT1v1 activated MEK and ERK as well as P38MAPK and JNK and sensitized cells to sorafenib and lenvatinib (Figure 4C,D and Figure S1C). The activation of MEK and ERK by SOD2 knockdown was higher in HuH6 cells than in HLF cells, similar to NEAT1 knockdown. In addition, it concomitantly suppressed AKT and conferred resistance to capivasertib (Figure 4E,F). However, mTOR and AMPK were not affected by SOD2 knockdown (Figure S1C). These results suggested that SOD2 switches the growth modalities of liver cancer cells downstream of NEAT1v1.

### 3.5. NEAT1v1 or SOD2 Knockdown Suppresses AKT Activity Independent of ER Stress

Unfolded protein accumulation in the endoplasmic reticulum (ER) causes ER stress, which triggers several pathways, including PERK/EIF2α, IRE1α/XBP1, and ATF6, to adapt to stress [23]. Upon increased ER stress, PERK is activated by self-phosphorylation and phosphorylates EIF2α. IRE1α, also activated by self-phosphorylation, executes XBP1 mRNA splicing, leading to XBP1 protein translation. ATF6 is activated by processing, and the p50ATF6 fragment translocates to the nucleus to initiate its target gene transcription. ER stress is also associated with the mTORC1/PI3K/AKT pathway [23]. Whereas, it was reported that SOD2 suppression by anticancer drugs increases oxidative stress, further aggravating ER stress [24]. These results implicate that SOD2 may regulate AKT activity through ER stress.

**Figure 5:**
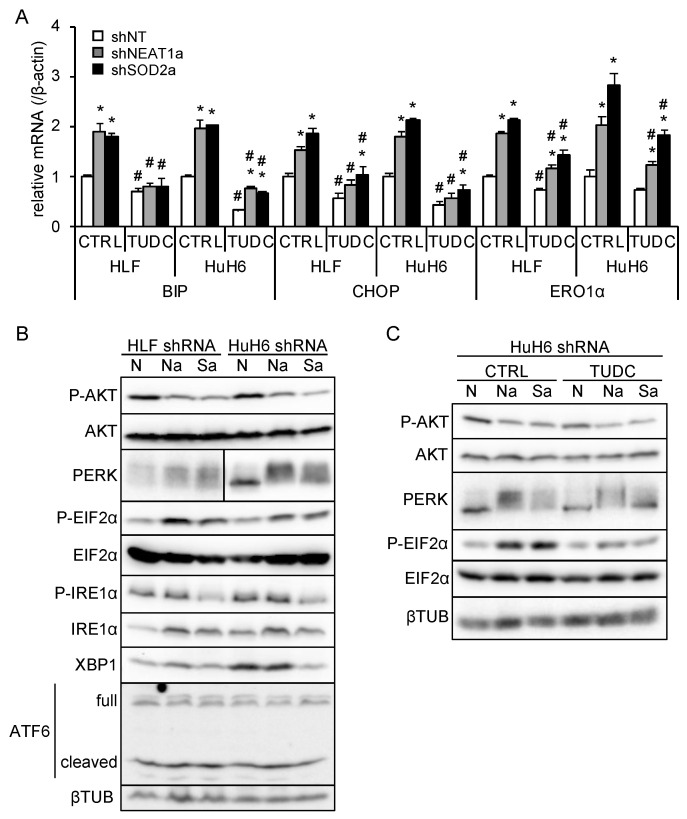
NEAT1v1 or SOD2 knockdown suppresses AKT activity independent of ER stress. (**A**) mRNA expression of ER stress target genes (BIP, CHOP, and ERO1α). HLF and HuH6 cell lines were transduced with adenoviruses expressing shNT, shNEAT1a, or shSOD2a in the presence of 0 mM (H2O; CTRL) or 2 mM TUDC for 48 h. * *p* < 0.05 vs. shNT; # *p* < 0.05 vs. CTRL (Tukey’s test; *n* = 3). (**B**) Representative Western blot images for the indicated proteins. βTUB is shown as an internal control. HLF and HuH6 cell lines were transduced with adenoviruses expressing shNT (N), shNEAT1a (Na), or shSOD2a (Sa) for 48 h. (**C**) Representative Western blot images for the indicated proteins. GAPDH is shown as an internal control. HuH6 cell lines were transduced with adenoviruses expressing shNT (N), shNEAT1a (Na), or shSOD2a (Sa) in the presence of 0 mM (H2O; CTRL) or 2 mM TUDC for 48 h.

ER stress induces the expression of its target genes, including binding-immunoglobulin protein (BIP), CCAAT/enhancer-binding protein homologous protein (CHOP), and ER oxidoreductase 1α (ERO1α), to ameliorate stress or induce apoptosis [23]. In liver cancer cell lines overexpressing NEAT1v1, these target genes, except for ERO1α, were downregulated (Figure S2A, Table S3). As previously observed [18], NEAT1v2 was upregulated only in HuH6 cells overexpressing NEAT1v1 (Figure S2A, Table S3). NEAT1 or SOD2 knockdown significantly increased BIP, CHOP, and ERO1α expression, whereas an ER stress inhibitor, TUDC, significantly suppressed their expression (Figure 5A and Figure S2B, Table S3). This result suggested that NEAT1 or SOD2 knockdown enhanced ER stress and that TUDC effectively counteracted it. In agreement with this, PERK was activated by NEAT1 or SOD2 knockdown, as indicated by an autophosphorylation-induced mobility shift and increased EIF2α phosphorylation (Figure 5B). Concomitantly, AKT activity was inhibited, as expected (Figure 5B). In contrast, the IRE1α/XBP1 pathway was suppressed by SOD2 knockdown, and ATF6 was unaffected by NEAT1 and SOD2 knockdown (Figure 5B). In agreement with the expression of ER stress target genes (Figure 5A), TUDC treatment suppressed PERK activation and EIF2α phosphorylation induced by NEAT1 and SOD2 knockdown (Figure 5C). However, the inhibition of AKT activity was not mitigated by TUDC (Figure 5C). These results suggested that NEAT1v1 or SOD2 knockdown suppresses AKT activity independent of ER stress.

## 4. Discussion

**Figure 6:**
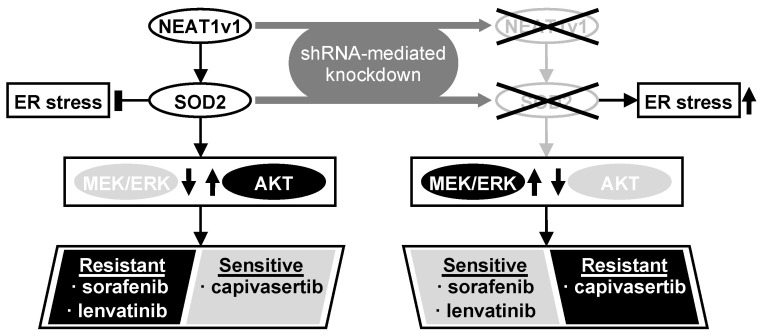
NEAT1v1 activates the AKT pathway through SOD2, thereby conferring sorafenib and lenvatinib resistance in liver cancer cells, which are concomitantly sensitized to capivasertib. This result suggests that NEAT1v1 switches the growth modality of liver cancer cells from MEK/ERK-dependent to AKT-dependent mode via SOD2. Consistently, NEAT1 or SOD2 knockdown results in MEK/ERK activation, thereby sensitizing liver cancer cells to sorafenib and lenvatinib and conferring capivasertib resistance. NEAT1v1 or SOD2 knockdown also exacerbates ER stress; however, AKT is suppressed in an ER stress-independent manner.

Although the precise function of NEAT1 in tumors is not fully clarified yet, NEAT1v1 plays important roles in HCC progression, such as the maintenance of liver CSCs and the acquisition of radioresistance through autophagy [17,18,19]. This study further elucidated that NEAT1v1 induces sorafenib and lenvatinib resistance, concomitantly with the suppression of MAPK signaling pathways and AKT activation through SOD2. Moreover, as a consequence of the AKT activation, NEAT1v1 sensitizes liver cancer cell lines to an AKT-targeted drug, capivasertib, suggesting that NEAT1v1 induces AKT addiction [25]. Although sorafenib and lenvatinib are clinically used for treating advanced HCC [2], their clinical efficacy is limited partly by the acquisition of drug resistance [7,26]. These results suggest that the NEAT1v1–SOD2 axis is one of the mechanisms underlying resistance to sorafenib and lenvatinib, as well as radiotherapy, and can be a therapeutic target and diagnostic marker for improving their clinical efficacy (Figure 6). Nonetheless, there are some limitations in our study that should be noted. First, all experiments in this study were performed in liver cancer cell lines; thus, the results must be further validated by in vivo studies. Second, HLF and HuH6 cell lines were established from HCC with mutations in the *TP53* gene and *telomerase reverse transcriptase* gene promoter of a 68-year-old male patient and hepatoblastoma with mutations in the *TP53* and *β-catenin* genes of a 1-year-old male patient, respectively [27,28]; thus, the pathological and pathogenic differences, and sex bias must be taken into consideration. Lastly, although the modulated expression of NEAT1v1 significantly changed cell viability, the changes are unlikely to be clinically significant under the current experimental conditions. This might be due to several reasons, such as a single-exposure and one-endpoint assessment, and an insufficient knockdown/overexpression efficiency. These weaknesses should be addressed by optimizing drug treatment conditions and employing knockout/rescue cell lines. Moreover, repeated exposure and continuous observation and assessment of tumors in in vivo models would show a more clinically significant effect than in vitro. However, a more important finding of this study is that NEAT1v1 is involved in the regulation of drug sensitivity in liver cancer cell lines by modulating growth signaling pathways.

We demonstrated that NEAT1 or SOD2 knockdown concomitantly activates P38MAPK and JNK in addition to MEK and ERK, while it was reported that P38MAPK can directly activate MEK in a RAF-independent manner [8]. However, in contrast to our results, this P38MAPK-induced MEK activation rendered HCC cells resistant to sorafenib [8]. Moreover, P38MAPK and JNK phosphorylation was not affected by NEAT1v1 overexpression, suggesting that these MAPK signaling molecules are unlikely to be involved in the mechanism underlying the drug resistance induced by the NEAT1v1–SOD2 axis. Based on these results, it is postulated that the NEAT1v1–SOD2 axis endows liver cancer cells with MEK/ERK-independent and AKT-dependent cell growth. Although this notion likely explains how NEAT1v1 lowered sensitivity to sorafenib and lenvatinib, more precise studies are needed.

The targets of sorafenib are VEGFR, PDGFR, FLT3, c-KIT, RAF1, and B-RAF [3,4], whereas lenvatinib inhibits VEGFR, PDGFR, RET, c-KIT, and FGFR [4,5,6]. Because RAF1 and B-RAF heterodimers phosphorylate MEK [29], it is thought that sorafenib preferentially inhibits the MAPK pathway [30]. In agreement with these findings, AKT activation is one of the mechanisms underlying the acquisition of sorafenib resistance in HCC [9,10,11,12,13,14]. Moreover, another group also reported that NEAT1 activates AKT via c-MET in HCC [20]. Although the relation between SOD2 and c-MET remains unclear, it may be worth studying it from the viewpoint of oxidative stress. In contrast, because receptor-type tyrosine kinase members transduce an extracellular signal to the MAPK and PI3K/AKT pathways, lenvatinib inhibits both pathways [4,6,31]. However, the mechanisms underlying lenvatinib resistance are currently not well understood. It was reported that the activities of AKT and ERK decreased and increased, respectively, in thyroid cancer cells treated with lenvatinib [32]. A MEK inhibitor, selumetinib, enhanced the cytotoxic effects of lenvatinib [32], suggesting that thyroid cancer cells switch cell growth modalities from AKT-dependent to MEK/ERK-dependent mode to acquire resistance against lenvatinib. Therefore, molecular switches between the two modes would provide important insights into lenvatinib resistance.

It is suggested that ER stress is involved in the regulation of AKT [23]. The suppression of SOD2 activity by anticancer drugs increased ER stress via oxidative stress, thereby inhibiting AKT, leading to apoptosis in HeLa cells [24]. Interestingly, SOD2 suppression concomitantly activates ERK and inhibits P38MAPK and JNK [24]. In contrast, an herbicide, paraquat, increases ER stress and activates AKT via PERK to promote epithelial-to-mesenchymal transition in pulmonary epithelial cells [33]. A VEGFR2-targeted drug, apatinib, was also shown to activate AKT via IRE1α activated by ER stress in esophageal squamous cell carcinoma [34]. Thus, AKT regulation by ER stress likely depends on the cellular context [23]. In liver cancer cell lines, NEAT1 or SOD2 knockdown activates the PERK/EIF2α pathway. However, amelioration of ER stress by TUDC fails to restore AKT activity. These results indicate that the NEAT1v1–SOD2 axis regulates AKT activity independent of ER stress.

It was demonstrated that a hydrogen peroxide-producing enzyme, NADPH oxidase 4, activates AKT to promote the growth and metastasis of lung cancer [35]. Therefore, hydrogen peroxide produced by SOD2 could be involved in the activation of AKT in liver cancer cells overexpressing NEAT1v1. Moreover, a genome-wide screen using a CRISPR/Cas9 library identified that Kelch-like ECH-associated protein 1 deficiency conferred resistance to sorafenib and lenvatinib in HCC cells [36]. Mechanistically, KEAP1 deficiency induced the activation of nuclear factor erythroid 2-related factor 2, which decreased sorafenib- and lenvatinib-induced oxidative stress through the upregulation of antioxidative stress factors [36]. These findings suggest that oxidative stress might play a central role in NEAT1v1-induced chemoresistance. The clarification of the underlying mechanism may provide a novel target for treating advanced HCC.

The recurrence of tumors is still a serious clinical problem, especially for systemic chemotherapy for advanced HCC. The data based on the WST assay demonstrated significant but modest sensitizing effects of NEAT1v1 knockdown to capivasertib, suggesting that the efficiency of NEAT1v1 knockdown must be improved to achieve clinically significant efficacy. In terms of this viewpoint, the recent successes of clinical trials using siRNAs or antisense oligonucleotides that target hepatocyte RNAs [37,38,39] make it attractive to establish NEAT1v1-targeting therapy in combination with capivasertib. Pre-clinical studies using in vivo models will provide more clinically important information for the development of a next-generation therapy for advanced HCC.

## 5. Conclusions

The NEAT1v1–SOD2 axis switches the growth modality from MEK/ERK- to AKT-dependent mode in male HCC and hepatoma cell lines and confers sorafenib and lenvatinib resistance. NEAT1v1 concomitantly sensitizes the liver cancer cell lines to an AKT-targeted drug, capivasertib. These findings would provide valuable clues to enhance the efficacy of sorafenib and lenvatinib treatment. Moreover, AKT would be a promising target for novel drugs for advanced HCC treatment.

## Data Availability

The raw data are available upon request, please contact the corresponding author.

## Supplementary Materials

The following are available online at https://www.mdpi.com/article/10.3390/cimb45020071/s1.
