# Supplementary material for: NEAT1–SOD2 Axis Confers Sorafenib and Lenvatinib Resistance by Activating AKT in Liver Cancer Cell Lines
Source: Curr Issues Mol Biol. 2023 Jan 29;45(2):1073–85. doi: 10.3390/cimb45020071 (PMC9955465; doi:10.3390/cimb45020071)
Supplement: Supplementary file 1 [file cimb-45-00071-s001.zip › Table S1.pdf]

Table S1: Key Resources Table

Reagents / Resource	Source	Identifier
**Cell Lines**		
HLF	Japanese Collection of Research Bioresources Cell Bank	JCRB0405
HuH6	Japanese Collection of Research Bioresources Cell Bank	JCRB0401
HLF overexpressing NEAT1v1	Our group	N/A
HuH6 overexpressing NEAT1v1	Our group	N/A
293A	Invitrogen	R70507
**Plasmid DNAs and vectors**		
pcDNA6-hNEAT1v1-AcGFP	Our group	N/A
pENTR/U6-AmCyan1	Our group	N/A
pAd/BLOCK-iT-DEST	Invitrogen	V49220
Adenovirus shNT	Our group	N/A
Adenovirus shNEAT1a/b	Our group	N/A
Adenovirus shSOD2a/b	Our group	N/A
**Chemicals and reagents**		
Ligation High version 2	Toyobo	LGK-201
Lipofec-tAMINE2000	Invitrogen	11668019
Blasticidin S, Hydrochloride	Kaken Pharmaceutical	KK-400
Sorafenib	Adipogen Life Sciences	AG-CR1-0025-M001
Lenvatinib mesylate	Toronto Research Chemicals	L215980
Capivasertib	Adooq Bioscience	A11759
Cell Counting Kit-8	Dojindo	CK04
Sodium tauroursodeoxycholate	Nacalai	32731-24
**Antibodies**		
AKT	Cell Signaling Technology	#9272
P-AKT (S473)	Cell Signaling Technology	#9271
P-AMPKα (T172)	Cell Signaling Technology	#4188
P-EIF2α (S51)	Cell Signaling Technology	#9721
P-ERK1/2 (Y202/204)	Cell Signaling Technology	#9101
IRE1α	Cell Signaling Technology	#3294
JNK	Cell Signaling Technology	#9252
P-JNK (T183/Y185)	Cell Signaling Technology	#9251
P-MEK1/2 (Ser217/221)	Cell Signaling Technology	#9154
P-mTOR (S2448)	Cell Signaling Technology	#2971
P38	Cell Signaling Technology	#9212
P-P38 (T180/Y182)	Cell Signaling Technology	#9211
AMPKα1/2	Santa Cruz Biotechnology	sc-74461
ATF4	Santa Cruz Biotechnology	sc-390063
ATF6α	Santa Cruz Biotechnology	sc-166659
EIF2α	Santa Cruz Biotechnology	sc-133132
ERK1/2	Santa Cruz Biotechnology	sc-514302
GAPDH	Santa Cruz Biotechnology	sc-365062
MEK1/2	Santa Cruz Biotechnology	sc-81504
PERK	Santa Cruz Biotechnology	sc-377400
βTUB	Santa Cruz Biotechnology	sc-55529
XBP1	Santa Cruz Biotechnology	sc-8015
P-IRE1(S724)	Abcam	ab124945
