# Supplementary material for: NEAT1–SOD2 Axis Confers Sorafenib and Lenvatinib Resistance by Activating AKT in Liver Cancer Cell Lines
Source: Curr Issues Mol Biol. 2023 Jan 29;45(2):1073–85. doi: 10.3390/cimb45020071 (PMC9955465; doi:10.3390/cimb45020071)
Supplement: Supplementary file 1 [file cimb-45-00071-s001.zip › Table S2.pdf]

Table S2: Oligo DNAs used in this study

**Oligo DNAs for adenovirus vector construction**	
shNT	
shNT-U	CACCGGGCGCGATAGCGCTAATAATTTCTCGAGAAATTATTAGCGCTATCGCGCTTT
shNT-D	GAAAAAAGCGCGATAGCGCTAATAATTTCTCGAGAAATTATTAGCGCTATCGCGCCC
shNEAT1a	
shNEAT1a-U	CACCGGGGAGTCGGTATTGTTGGTAATCTCGAGATTACCAACAATACCGACTCCTTT
shNEAT1a-D	GAAAAAAGGAGTCGGTATTGTTGGTAATCTCGAGATTACCAACAATACCGACTCCCC
shNEAT1b	
shNEAT1b-U	CACCGGATGGACCGTGGTTTGTTACTACTCGAGTAGTAACAAACCACGGTCCATTTT
shNEAT1b-D	GAAAAAAATGGACCGTGGTTTGTTACTACTCGAGTAGTAACAAACCACGGTCCATCC
shSOD2a	
shSOD2a_U	CACCGGGCACGCTTACTACCTTCAGTACTCGAGTACTGAAGGTAGTAAGCGTGCTTT
shSOD2a_D	GAAAAAAGCACGCTTACTACCTTCAGTACTCGAGTACTGAAGGTAGTAAGCGTGCCC
shSOD2b	
shSOD2b_U	CACCGGGTGGTGGTCATATCAATCATACTCGAGTATGATTGATATGACCACCACTTT
shSOD2b_D	GAAAAAAGTGGTGGTCATATCAATCATACTCGAGTATGATTGATATGACCACCACCC

**qPCR primers**	
Adenovirus titration	
AdE2B_Titer_For	TGTCAAGCTTGGTGGCAAAC
AdE2B_Titer_Rev	TCGCGACAAAAACCAAACCC
mRNA determination	
ACTB_S	GATGCAGAAGGAGATCACTGC
ACTB_AS	TGATCCACATCTGCTGGAAG
total NEAT1_S	TGCCACAACGCAGATTGATG
total NEAT1_AS	ACAAGAAGGCAGGCAAACAG
NEAT1v2_S	AGGGTTCTGTTGCTAACACG
NEAT1v2_AS	TGCCGATGAAGCAACAAAGC
SOD2_S	ACATCAACGCGCAGATCATG
SOD2_AS	TGCAGGCTGAAGAGCTATCTG
BIP_S	AACCGCTGAGGCTTATTTGG
BIP_AS	TCTTTGGTTGCTTGGCGTTG
CHOP_S	GCGCATGAAGGAGAAAGAACAG
CHOP_AS	ATTTCCTGCTTGAGCCGTTC
ERO1α_S	AAGGGACTGTGCTGTCAAAC
ERO1α_AS	CTCCAAGTCGTTCAGCTTGTTC
