# Supplementary material for: NEAT1–SOD2 Axis Confers Sorafenib and Lenvatinib Resistance by Activating AKT in Liver Cancer Cell Lines
Source: Curr Issues Mol Biol. 2023 Jan 29;45(2):1073–85. doi: 10.3390/cimb45020071 (PMC9955465; doi:10.3390/cimb45020071)
Supplement: Supplementary file 1 [file cimb-45-00071-s001.zip › Table S3.pdf]

Table S3: Ct values [*1]

Figure 5A			β-actin	BIP	CHOP	ERO1α
HLF	CTRL	shNT	15.80	17.11	21.96	21.72
		shNEAT1a	15.67	16.05	21.21	20.69
		shSOD2a	15.98	16.43	21.25	20.81
	TUDC	shNT	15.29	17.14	22.24	21.67
		shNEAT1a	15.30	16.93	21.71	21.02
		shSOD2a	15.65	17.27	21.75	21.06
HuH6	CTRL	shNT	15.75	18.01	22.84	21.94
		shNEAT1a	15.65	16.93	21.89	20.80
		shSOD2a	16.19	17.43	22.19	20.87
	TUDC	shNT	15.35	19.12	23.64	21.98
		shNEAT1a	15.13	17.78	23.01	21.00
		shSOD2a	15.74	18.60	23.25	21.06
Figure S3A			β-actin	BIP	CHOP	ERO1α
	HLF	CTRL	13.68	16.10	18.20	20.34
		NEAT1v1	13.70	16.90	19.17	20.19
	HuH6	CTRL	13.43	16.75	19.76	20.39
		NEAT1v1	13.59	18.04	22.21	20.72
Figure S3B			total NEAT1	NEAT1v2	SOD2	
HLF	CTRL	shNT	21.18	22.74	23.02	
		shNEAT1a	21.72	23.30	23.27	
		shSOD2a	21.38	23.06	24.58	
	TUDC	shNT	21.05	22.68	22.44	
		shNEAT1a	21.81	23.10	22.76	
		shSOD2a	21.06	22.96	24.15	
HuH6	CTRL	shNT	21.98	23.49	21.42	
		shNEAT1a	22.50	23.93	21.61	
		shSOD2a	22.35	24.06	22.84	
	TUDC	shNT	21.79	23.17	21.04	
		shNEAT1a	22.64	24.09	21.60	
		shSOD2a	22.21	23.62	22.37	

*1. Values are means of 3 experiments.
