# Supplementary figures and images for: NEAT1–SOD2 Axis Confers Sorafenib and Lenvatinib Resistance by Activating AKT in Liver Cancer Cell Lines
Source: Curr Issues Mol Biol. 2023 Jan 29;45(2):1073–85. doi: 10.3390/cimb45020071 (PMC9955465; doi:10.3390/cimb45020071)

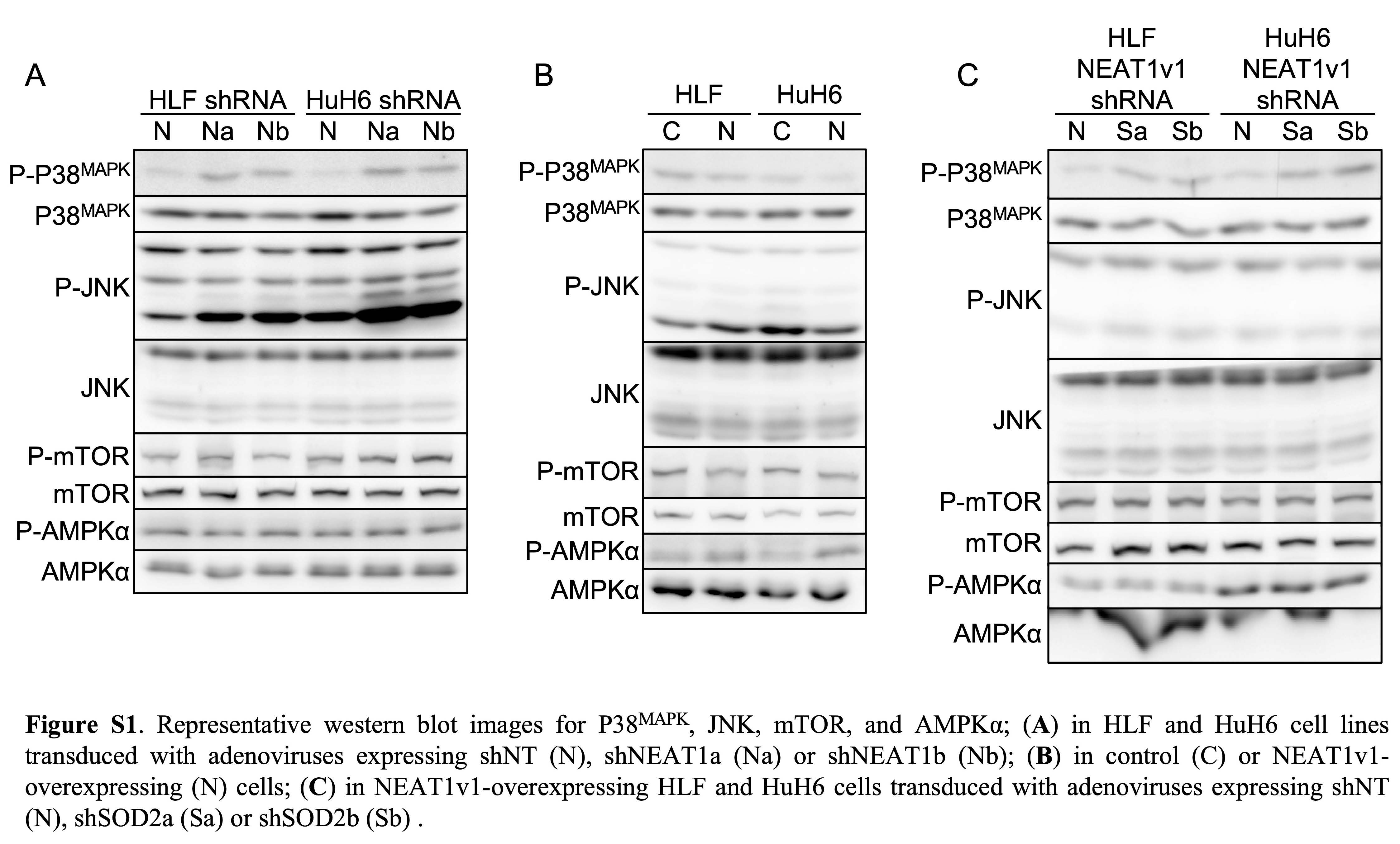

Supplement: Supplementary file 1 [file cimb-45-00071-s001.zip › Figure S1.jpg]

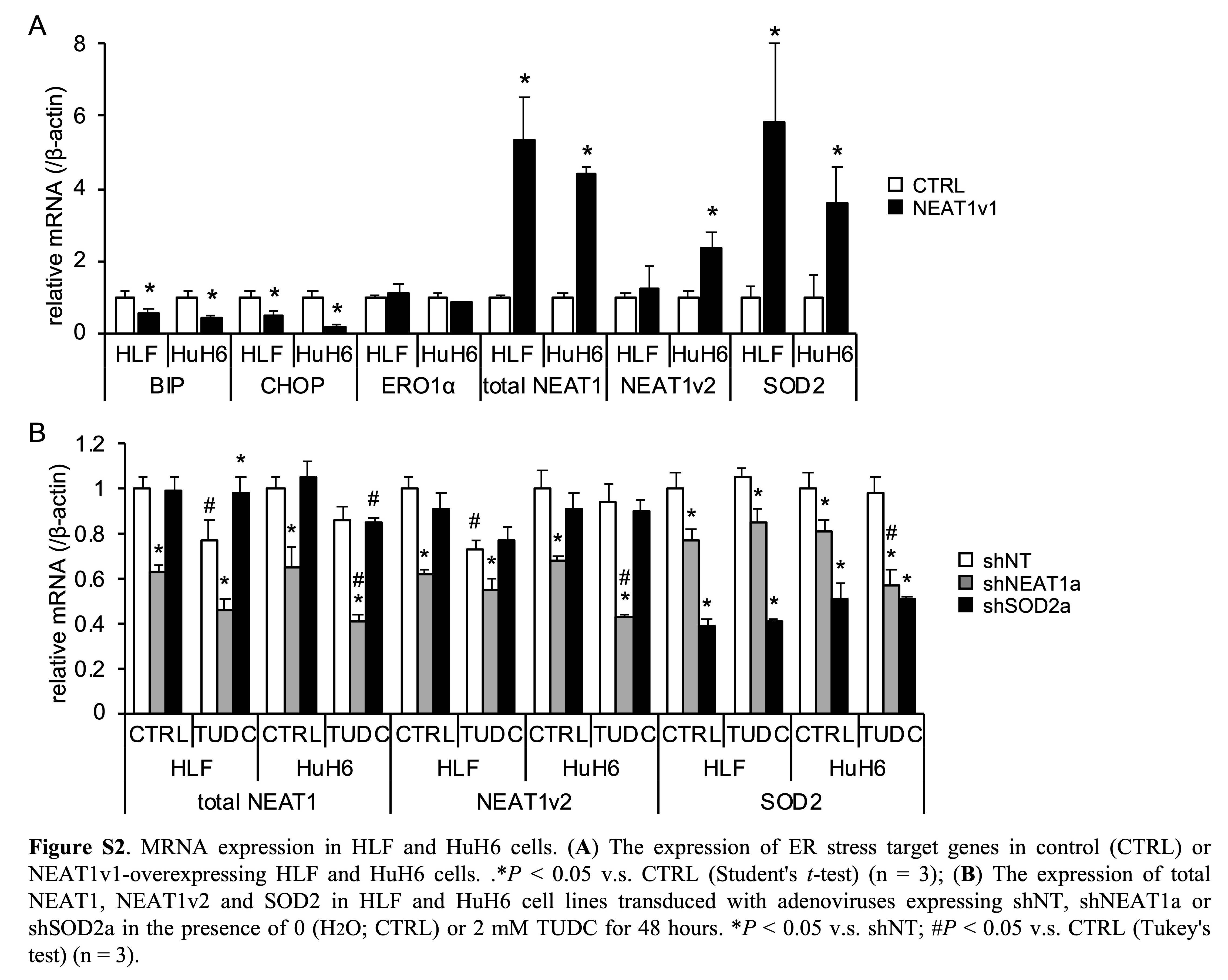

Supplement: Supplementary file 1 [file cimb-45-00071-s001.zip › Figure S2.jpg]
